# Prevalence of temporomandibular joint disorder in the Lebanese population, and its association with depression, anxiety, and stress

**DOI:** 10.1186/s13005-020-00234-2

**Published:** 2020-09-04

**Authors:** Elio Kmeid, Mansour Nacouzi, Souheil Hallit, Ziad Rohayem

**Affiliations:** 1 Faculty of Medicine and Medical Sciences, Holy Spirit University of Kaslik (USEK), Jounieh, Lebanon; 2 Department of otorhinolaryngology, Eye and Ear Hospital, Naccache, Lebanon; 3 INSPECT-LB: Institut National de Santé Publique, Épidémiologie Clinique et Toxicologie- Liban, Beirut, Lebanon

**Keywords:** Temporomandibular joint disorder, Depression, Anxiety, Stress

## Abstract

**Background:**

The objectives of this study were to study the prevalence of temporomandibular joint disorder (TMD) and its association with anxiety, depression, and stress among the general Lebanese population as well as in a sample of patients recruited from an otolaryngologist clinic.

**Methods:**

A cross-sectional study was conducted between September 2018 and December 2019, which enrolled 459 participants from all districts of Lebanon (sample 1) and 37 patients from the otolaryngologist clinic at the Eye and Ear Hospital (sample 2). The temporomandibular disorder screening checklist was used to screen for temporomandibular joint disorder. The Fonseca’s anamnestic index was used to assess for temporomandibular joint disorder related signs and symptoms, as well as for symptoms severity.

**Results:**

The results showed that 19.7% of the general Lebanese population had TMD, from which 55.9% were female. In contrast, 59.5% of patients in the sample recruited from the clinic were found to have TMD. Higher stress, anxiety, and depression scores were associated with higher temporomandibular disorder severity score (B = 0.23; B = 0.10 and B = 0.10 respectively). Patients in the sample recruited from the clinic had higher mean stress (20.75 vs 11.43), anxiety (12.46 vs 5.78), depression (13.24 vs 6.52), and temporomandibular disorder severity scores (59.5% vs 19.7%) than the general population.

**Conclusion:**

Temporomandibular joint disorder appears to be associated significantly with depression, anxiety, and stress and remains largely underdiagnosed in the general population.

## Background

Temporomandibular joint disorder is a group of pain conditions that affect the function of the temporomandibular joint, along with the muscles of mastication [1, 2]. Pain in the area around the temporomandibular joint, can be due to diseases from inside the articulation, from adjacent structures, or from a combination of both [3]. There may be associated symptoms, not related to the musculoskeletal system, like tinnitus, referred otalgia, headaches (tension headache or migraine), toothache, neck pain, and myofascial pain [4].

Myofascial TMD pain is the most frequent cause of orofacial pain (42%), followed by disc displacement with reduction (32.1%) and arthralgia (30%) [5]. Myofascial Pain is defined as “pain of muscle origin that is affected by jaw movement, function, or para-function, and replication of this pain occurs with provocation testing of the masticatory muscles spreading beyond the site of palpation but within the boundary of the muscle when using myofascial examination protocol” [6]. In respect to TMD, the term Masticatory Myofascial Pain (MTMD) can be used to describe the myogenic component of the disease.

Temporomandibular joint disorder can affect 5 to 12% of the population [6]. Some studies have even reported higher incidences up to 25% [7] and 33% [8] to 40% [9] in the general population. Less than 5% of patients will seek medical treatment [8]. Whereas some patients will more likely seek dental care for their temporomandibular joint symptoms [10]. A systematic review conducted by Lai et Al. has shown the prevalence of TMD among orthodontic patients to range from 21.1 to 73.3% [11]. According to a World Health Organization (WHO) report, TMD is the third stomatological disorder, after dental caries and periodontal diseases, to be considered a populational disease [12]. The symptoms of temporomandibular joint dysfunction are more common in the female population, compared to males [13–16]. Mcfarlane et Al. (2002) have stated that the prevalence of orofacial pain in temporomandibular joint disorder was 21 and 30% in males and females, respectively. Young females less than 30 years old are at increased risk of temporomandibular joint disorder [17]. In contrast to the previous reports, some recent studies have shown that temporomandibular joint disorder prevalence reaches its peak between 45 to 64 years of age, before decreasing with older age as older adults seemed to have milder symptoms of temporomandibular joint disorder [18].

The etiologies of temporomandibular joint disorder are biologic, environmental (smoking), emotional (depression and anxiety), social, and cognitive factors. There is a constant association with other pain conditions (like chronic headaches), fibromyalgia, autoimmune disorders (like Sjogren syndrome, rheumatoid arthritis, and lupus erythematosus), psychiatric illness, and sleep apnea [17]. One study has shown an association between temporomandibular joint disorder symptoms and depression, anxiety, oral parafunctions especially bruxism, and hysteria in adolescents [19]. Another one has shown that two important risk factors temporomandibular joint were sleep quality and stress level [20].

Temporomandibular joint disorder has also been shown to be associated with impaired general health status and socioeconomic factors in Sweden (such as higher education level, university degree and chewing with caution) [21]. Another study has shown that increased level of pain has been associated with lower educational level, divorce or separation, and female gender [22]. Racial disparities have also been described as facial and jaw pain were shown to be more frequent among Caucasians, with an earlier onset, compared to African-Americans [23].

People with temporomandibular joint disorder are more commonly affected by anxiety and depression [24]. Civil war in Lebanon has increased the risk of mental health disorders [25]. However, because there is a significant delay in seeking treatment for mental health, mental disorders are underreported [25]. There seems to be a lack of data regarding the prevalence of temporomandibular joint disorder in Lebanon in general and Masticatory Myofascial Pain (MTMD) in particular and its association with levels of anxiety, stress and depression as well as oral parafunctional habits. Therefore, the objectives of this study were to study the prevalence of temporomandibular joint disorder (TMD) and its association with anxiety, depression, and stress among the general Lebanese population as well as in a sample of patients recruited from an otolaryngologist clinic.

## Methods

### Study 1

#### Study design

**Fig. 1:**
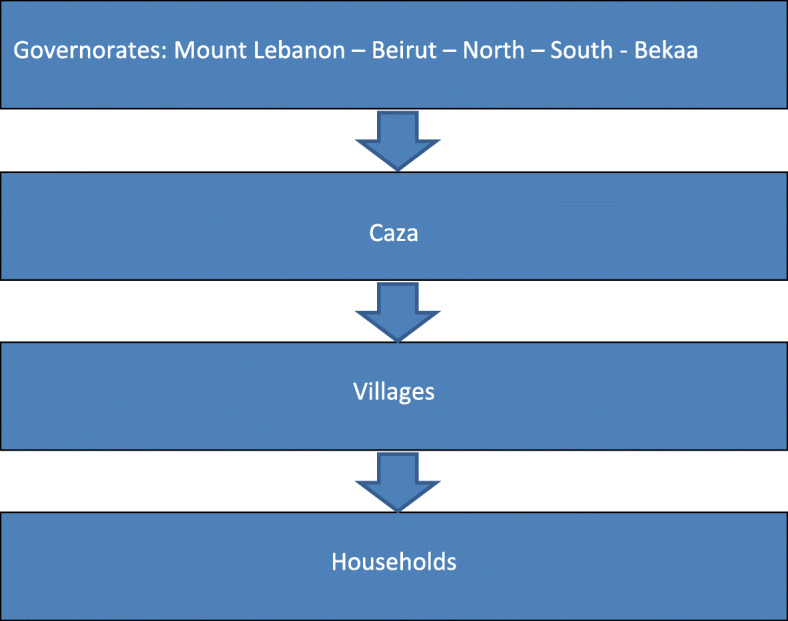
Diagrammatic representation for participant selection

Between September 2018 and December 2019, a proportionate sample from all Lebanese governorates was collected. Each governorate is divided into Caza, which in turn, is divided into a variety of villages (Fig. 1). A simple randomization technique was used to choose two villages and the households from each village. All adults persons in the household were invited to participate. Questionnaires were filled by those who accepted enrollment in the study. Exclusion criteria included patients with congenital craniofacial malformations, reported history of facial trauma, history of TMJ surgery, history of rheumatological or autoimmune disorder, as these are well known causes of TMD [17, 26], and a recent (< 6 weeks) dentists visit, raising suspicion of dental disease which can mimic symptoms of TMD [17, 26]. In addition, people already taking medications for depression, anxiety, and stress, were also excluded, as the score severity of each of depression, anxiety, and stress might be affected and lowered by these.

#### Minimal sample size calculation

Based on the formula $\boldsymbol{n}=\frac{{\left({\boldsymbol{Z}}_{\mathbf{1}-\boldsymbol{\alpha} /\mathbf{2}}\right)}^{\mathbf{2}}\boldsymbol{p}\left(\mathbf{1}-\boldsymbol{p}\right)}{{\boldsymbol{d}}^{\mathbf{2}}}$, where *n* = size of the sample, *p* = expected proportion and d = the desired margin of error and *Z*_1 − *α*/2_ = 1.96 for *α* = 5%, a minimal sample of 384 participants was needed, based on a *p* = 50% expected frequency of TMJ in the absence of similar studies in the country and a d = 5% risk of error; 510 questionnaires were distributed, and 443 (86.86%) were collected back.

#### Translation

A translation procedure from English to Arabic of all non-validated scales was made by one specialized translator, then back to English by a different translator. Then, a comparison of the two English versions was made to make sure that no contradictions exist between the 2 versions. Discrepancies were resolved by consensus. A pilot study on 20 participants was first conducted; no significant changes were made to the questionnaire content, therefore, these patients were included in the final database.

#### Questionnaire

The questionnaire used was in two languages, the English language and the translated version to the native language of Lebanon (Arabic), and left for the participant preference. The first part of the questionnaire assessed the sociodemographic characteristics of the included participants (age, gender, education level, socioeconomic level, sociodemographic level, weight, height), and the other part consisted of the different scales used in this study:

#### Temporomandibular disorder screening checklist

This short and high validity screening checklist goal, is for primary TMD screening in general practice [27]. TMD screening is of practical importance in dentistry. Plus, it could be of use in general population settings to determine one’s capabilities to execute some tasks, like in military, where personnel are to be deployed and moved to areas where a treatment for acute pain episodes will be difficult [28].

#### Fonseca’s anamnestic index

It consisted of measuring the TMD severity using the FAI questionnaire. It is composed of 10 questions with three answer options, each one of them assigned to a score: yes = 10 points, sometimes = 5 points and no = 0. According to the total results obtained to each individual, subjects were categorized to no TMD (0–3), mild TMD (5–9), moderate TMD (10–14), or severe TMD (15–19) [29, 30].

#### PHQ-9 questionnaire

It consisted of nine items assessing depression and its severity: mild, moderate, moderately severe, and severe depression, represented by cutoffs values of 5, 10, 15, and 20, respectively [31].

### GAD-7

It aimed at screening for general anxiety disorder. It has been demonstrated as an effective and valid tool for measuring its severity practically and in the research setting. Higher scores reflect higher anxiety [32]. Both PHQ-9 and GAD-7 scales have been previously validated in Lebanon [33].

#### Beirut Distress Scale (BDS22)

Developed and validated in Lebanon, this scale assesses mental distress, with higher scores indicative of higher psychological distress) [34].

### Study 2

In addition, a random sample of adult patients coming to an otolaryngologist clinic with head and neck symptoms were selected. Adults (18 years old and more) were eligible to participate. The exact same exclusion criteria were applied to sample 2. More specifically, patients previously diagnosed or treated for TMD were excluded. The purpose of sample two was to present a “real-life scenario” alternative to sample 1. The hypothesis was to see whether patients presenting to ENT clinic suffering from any ENT-related complaint had a higher incidence of TMD than the general population. This was based on the impression that patient with TMD tend to visit otolaryngologists more frequently than dentists, neurologists or rheumatologists because they tend to present frequently with referred otalgia. This raises the point of lack of awareness among patients and physicians alike on the common presentation and screening of TMD. As for sample 1, screening of TMD was solely based on anamnesis and findings on physical exam were not included in the analysis. This was done purposely to apply same diagnostic criteria among the two sample as the main purpose of sample 2 as previously noted was to compare incidence of undiagnosed-untreated TMD among the 2 samples.

### Statistical analysis

Statistical Package for Social Science (SPSS) version 23 was used for the statistical analyses. Weighting to the general population was performed in terms of age, gender, and mouhafaza. Descriptive statistics were presented using mean and standard deviation for continuous measures, frequencies and percentages for categorical variables. The Student t-test and ANOVA test were used to assess the association between each continuous independent variable (PHQ-9, GAD-7 and BDS22 scores) and the sociodemographic and other variables. To calculate the *p*-value of the statistical significance, the Bonferroni correction compensates for that increase by testing each individual hypothesis at a significance level of α/m, where α is the desired overall alpha level and m is the number of hypotheses/tests conducted (24). Concerning the knowledge, attitude and practice scores, we tested 27 hypotheses/variables in each model, with a desired error α of 0.05; therefore, the Bonferroni correction would test each individual hypothesis at a *p*-value of 0.05/27 = 0.002. Multivariable linear regression models were done to explore factors associated with the three scores as dependent variables and taking all variables that showed a *p* ≤ 0.002 in the bivariate analysis as independent variables. A *p* < 0.05 in the multivariable model was considered significant. Scales’ reliability was assessed using Cronbach’s alpha.

## Results

High Cronbach’s alpha values were obtained for all the scales as follows: FAI (0.783), PHQ-9 (0.831), GAD-7 (0.868) and BDS22 (0.923).

### Study 1

#### Sociodemographic and other characteristics

The results of the sociodemographic characteristics are summarized in Table 1. The mean age of the participants was 30.88 ± 14.50 years. 54.7% were of female gender. 87 (19.7%) of the participants had TMJ [95% CI 0.160–0.234], with a mean TMD severity (FAI score) of 22.02 ± 17.50. 55.9% of patients with TMD were female. The mean depression score was 6.51 ± 4.51, anxiety 5.78 ± 4.04 and the mean stress score was 11.43 ± 9.40.

**Table 1:** Sociodemographic characteristics of the participants (*N* = 443)

Variable	N (%)
**Gender**	
Male	196 (45.3%)
Female	237 (54.7%)
**Mouhafaza**	
Beirut	43 (10.6%)
Mount Lebanon	156 (38.6%)
North	77 (19.1%)
South	52 (12.9%)
Bekaa	76 (18.8%)
**Education level**	
Illiterate/primary/complementary	18 (4.2%)
Secondary	168 (38.8%)
University	247 (57.0%)
**Monthly income**	
< 1000 USD	106 (34.3%)
1000–2000 USD	135 (43.7%)
> 2000 USD	68 (22.0%)
**Mean ± SD**	
Age (in years)	30.88 ± 14.50
Body Mass Index (Kg/m^2^)	24.27 ± 6.94

Pain in the jaw was the most reported symptoms (69.1%) followed by pain in the ear (50.5%), pain in front of the ear (43.6%), pain in the temple (42.1%) and finally facial pain (36% of the patients).

#### Bivariate analysis

Tables 2 and 3 summarize the results of the bivariate analysis of variables associated with depression, anxiety and stress. A higher depression score was found to be significantly associated with bruxism at night compared to not. A higher number of working hours per day was significantly associated with higher depression, whereas a higher number of hours on the phone per day was correlated with higher TMD severity and higher depression, anxiety and stress. Higher age was significantly associated with lower depression and stress.

**Table 2:** Bivariate analysis of factors associated with depression, anxiety and stress

Variable	Depression	Anxiety	Stress
**Gender**			
Male	5.92 ± 4.17	5.10 ± 3.49	10.24 ± 8.44
Female	6.97 ± 4.75	6.32 ± 4.34	12.41 ± 10.11
*p*	0.035	0.007	0.042
**Governorate**			
Beirut	6.23 ± 4.43	6.67 ± 3.29	9.53 ± 9.54
Mount Lebanon	6.81 ± 4.60	6.06 ± 4.28	12.42 ± 9.76
North	6.26 ± 5.12	5.40 ± 4.07	11.14 ± 9.68
South	6.67 ± 4.91	6.11 ± 4.39	11.52 ± 8.70
Bekaa	6.03 ± 3.69	4.87 ± 3.36	10.49 ± 8.88
*p*	0.729	0.065	0.243
**Education level**			
Illiterate/primary/ complementary	4.22 ± 3.93	4.39 ± 3.96	8.17 ± 8.31
Secondary	6.80 ± 4.81	5.91 ± 4.24	12.97 ± 10.26
University	6.47 ± 4.32	5.73 ± 3.91	10.44 ± 8.72
*p*	0.074	0.330	0.018
**Monthly income**			
< 1000 USD	6.47 ± 4.42	5.23 ± 3.48	11.16 ± 8.68
1000–2000 USD	6.38 ± 4.36	5.81 ± 4.10	10.52 ± 9.19
> 2000 USD	5.83 ± 4.26	5.13 ± 3.30	10.19 ± 8.21
*p*	0.617	0.510	0.684
**Sleep apnea**			
No	6.35 ± 4.27	5.59 ± 3.75	10.98 ± 8.90
Yes	7.97 ± 5.40	7.36 ± 5.07	14.65 ± 11.57
*p*	0.036	0.014	0.027
**Cigarette smoking**			
No	6.43 ± 4.44	5.73 ± 4.02	11.12 ± 9.01
Yes	7.09 ± 4.88	6.09 ± 4.18	13.43 ± 11.23
*p*	0.364	0.667	0.264
**Waterpipe smoking**			
No	6.49 ± 4.56	5.79 ± 4.04	11.22 ± 9.22
Yes	6.73 ± 4.25	5.76 ± 4.04	12.82 ± 10.29
*p*	0.503	0.931	0.278
**Bruxism at night**			
No	6.12 ± 4.34	5.53 ± 3.85	10.71 ± 8.81
Yes	7.91 ± 4.43	6.62 ± 4.08	14.09 ± 10.53
*p*	**0.001**	0.051	0.008
**Dental gutter**			
No	6.36 ± 4.53	5.75 ± 4.06	11.02 ± 9.31
Yes	7.75 ± 4.33	6.17 ± 3.84	14.18 ± 9.60
*p*	0.012	0.275	0.017
**Presence of TMJ**			
No	6.21 ± 4.41	5.50 ± 3.95	10.82 ± 9.04
Yes	7.77 ± 4.69	6.91 ± 4.19	13.88 ± 10.47
*p*	0.005	0.01	0.015
**Kind of work**			
Desk	7.43 ± 5.03	6.55 ± 4.42	13.04 ± 10.60
Standing	6.00 ± 4.24	5.61 ± 4.10	11.05 ± 8.66
Computer	5.97 ± 4.35	5.23 ± 3.39	9.33 ± 8.87
*p*	0.042	0.08	0.036

Numbers in bold indicate significant *p*-values based on the corrected *p* ≤ 0.002.

**Table 3:** Bivariate analysis of continuous variables associated with depression, anxiety and stress

Variable	Depression	Anxiety	Stress
Age	**−0.198**	−0.125	**− 0.184**
Body Mass Index	−0.044	− 0.114	−0.035
Working hours daily	**0.178**	0.153	0.047
Hours on phone daily	**0.199**	**0.154**	**0.215**
Disorder severity (FAI score)	**0.472**	**0.407**	**0.466**
Cumulative cigarette smoking	0.041	0.047	0.074
Cumulative waterpipe smoking	0.033	0.002	0.075

Numbers in bold indicate significant *p*-values based on the corrected p ≤ 0.002.

#### Multivariable analysis (presence vs absence of TMJ pain)

The results of a first linear regression, taking depression (PHQ-9 score) as the dependent variable, showed that having bruxism at night (B = 2.29) and a higher number of working hours per day (B = 0.21) were significantly associated with higher depression, whereas higher age (B = − 0.09) was significantly associated with lower depression (Table 4, Model 1).

**Table 4:** Multivariable analysis taking the depression, anxiety and stress scores as dependent variables and the presence/absence of TMD as an independent variable

**Model 1: Linear regression taking depression (PHQ-9 score) as the dependent variable.**					
**Variable**	**UB**	**SB**	***p***	**95% Confidence Interval**	
Age	-0.09	−0.26	< 0.001	− 0.12	− 0.05
Bruxism at night (yes vs no*)	2.29	0.20	< 0.001	1.13	3.44
Working hours per day	0.21	0.13	0.009	0.05	0.37
Presence vs absence* of TMD	0.92	0.08	0.137	−0.29	2.12
**Model 2: Linear regression taking anxiety (GAD-7 score) as the dependent variable.**					
**Variable**	**UB**	**SB**	***p***	**95% Confidence Interval**	
Hours on phone per day	0.18	0.18	< 0.001	0.08	0.28
Presence vs absence* of TMD	1.39	0.14	0.006	0.40	2.39
**Model 3: Linear regression taking stress (BDS22 score) as the dependent variable.**					
**Variable**	**UB**	**SB**	***p***	**95% Confidence Interval**	
Hours on phone per day	0.43	0.18	< 0.001	0.20	0.67
Age	−0.12	−0.17	0.001	−0.18	−0.05
Presence vs absence* of TMD	3.48	0.15	0.003	1.22	5.74

*Reference group; *UB* Unstandardized Beta, *SB* Standardized Beta

The results of a second linear regression, taking anxiety (GAD-7 scores) as the dependent variable, showed that the presence of TMD (B = 1.39) and a higher number of hours on the phone per day (B = 0.18) were significantly associated with higher anxiety (Table 4, Model 2).

The results of a third linear regression, taking stress (BDS22 scores) as the dependent variable, showed that the presence of TMD (B = 3.48) and a higher number of hours on the phone per day (B = 0.43) were significantly associated with higher stress, whereas higher age (B = -0.12) was significantly associated with lower stress (Table 4, Model 3).

#### Multivariable analysis (pain severity)

The results of a first linear regression, taking depression (PHQ-9 score) as the dependent variable, showed that a higher TMD score (B = 0.10), having bruxism at night (B = 1.12) and a higher number of working hours per day (B = 0.17) were significantly associated with higher depression, whereas higher age (B = − 0.06) was significantly associated with lower depression (Table 5, Model 1).

**Table 5:** Multivariable analysis taking the depression, anxiety and stress scores as dependent variables and the TMDseverity score as an independent variable

**Model 1: Linear regression taking depression (PHQ-9 score) as the dependent variable.**					
**Variable**	**UB**	**SB**	***p***	**95% Confidence Interval**	
TMD severity (FAI score)	0.10	0.40	< 0.001	0.08	0.13
Age	-0.06	−0.18	< 0.001	−0.09	− 0.03
Working hours per day	0.17	0.10	0.028	0.02	0.31
Bruxism at night (yes vs no*)	1.12	0.10	0.046	0.02	2.22
**Model 2: Linear regression taking anxiety (GAD-7 score) as the dependent variable.**					
**Variable**	**UB**	**SB**	***p***	**95% Confidence Interval**	
TMD severity (FAI score)	0.10	0.45	< 0.001	0.08	0.12
Hours on phone per day	0.13	0.13	0.005	0.04	0.22
**Model 3: Linear regression taking stress (BDS22 score) as the dependent variable.**					
**Variable**	**UB**	**SB**	***p***	**95% Confidence Interval**	
TMD severity (FAI score)	0.23	0.42	< 0.001	0.18	0.28
Hours on phone per day	0.37	0.15	< 0.001	0.15	0.58
Age	−0.07	−0.10	0.031	−0.13	− 0.01

*Reference group; *UB* Unstandardized Beta, *SB* Standardized Beta

Variables entered in the models: Model 1: Bruxism at night, age, FAI pain severity score, hours on the phone per day; Model 2: FAI pain severity score, hours on the phone per day; Model 3: FAI pain severity score, hours on the phone per day, age.

The results of a second linear regression, taking anxiety (GAD-7 scores) as the dependent variable, showed that a higher TMD severity score (B = 0.10) and a higher number of hours on the phone per day (B = 0.13) were significantly associated with higher anxiety (Table 5, Model 2).

The results of a third linear regression, taking stress (BDS22 scores) as the dependent variable, showed that a higher TMD severity score (B = 0.23) and a higher number of hours on the phone per day (B = 0.37) were significantly associated with higher stress, whereas higher age (B = -0.07) was significantly associated with lower stress (Table 5, Model 3).

### Study 2

#### Comparison between the sample from the general population and that recruited from the ENT clinic

Higher mean depression, anxiety, stress and TMD severity scores were significantly found in the sample recruited from the clinic compared to that from the general population (Table 6). Moreover, a significantly higher percentage of patients who came to the clinic had TMD compared to the general population (59.5% vs 19.7%; *p* < 0.001). These results indicate that 19.7% of TMD remain undiagnosed in the general population.

**Table 6:** Comparison between the sample from the general population and the clinic one

Variable	General population	Clinic	***p***
Depression	6.52 ± 4.51	13.24 ± 7.35	< 0.001
Anxiety	5.78 ± 4.03	12.46 ± 5.65	< 0.001
Stress	11.43 ± 9.40	20.75 ± 18.94	0.029
TMD severity (FAI score)	22.02 ± 17.50	59.86 ± 18.80	< 0.001

## Discussion

To the best of our knowledge, this is the first study to be conducted in Lebanon to target the prevalenc of temporomandibular joint disorder, and its association with anxiety, depression, and stress. Furthermore, no previously published studies have ever compared the severity of TMD, stress, anxiety, and depression between a sample recruited from the clinic, and another one from the general population.

### Analysis of the results

First, our study has shown that the prevalence of undiagnosed temporomandibular joint disorder among the Lebanese population is 19.7%. Moreover, people having a higher TMD severity score were more likely to have bruxism at night, and a higher number of working hours per day. They were also more likely to have higher depression scores, whereas older age was associated with lower depression and stress levels. The presence of TMD and having a higher TMD severity score was also associated with higher number of hours spent on the phone per day and higher anxiety and stress levels. In addition, a higher mean depression, anxiety, stress, and TMD severity scores were found in the clinic sample, compared to the general population sample. This correlated with the fact that a higher percentage of patients recruited from the clinic had a TMD, compared to the general population. Otolaryngologists are among the first-line physicians dealing with TMD. This could be explained by the relative high incidence of referred otalgia in the TMD group pushing patients to consult ENT to rule-out otitis.

### Study results compared to international studies

Our study has shown that the prevalence of TMD among the Lebanese participants was 19.7%. It was significantly higher than its prevalence in the US (4.6%) [35]. A study conducted on physicians in Saudi Arabia showed also a high prevalence of TMJ disorders (37%) [1]. TMD symptoms were also found to affect 39.2% of Brazilian population in a study conducted by Goncalves et al. [36]. When focusing on university students, the prevalence of TMD was 46.1% in Mexico [37], 42.9% in Taiwan [38], 49.7% in North Saudi University [39], and 53.21% [30] to 68% [40] in Brazil.

Signs and symptoms of TMD were more common in the female population, which comes in agreement with other studies [16, 41]. In our current study, we have found that the most common symptom of TMD was pain in the jaw (69.1%), as reported in other studies [41, 42]. In our study, 19.7% of patients with TMD remained undiagnosed, yet they presented sometimes the typical signs and symptoms of TMD, and sometimes even with a high severity scores. We have found a strong association between TMD and depression, as shown in other studies [43]. Anxiety was also associated with TMD in our study, which is in agreement with findings in another study made among university undergraduate students [44] and among pre-university students in relation to stress attributed to their university entrance exam [45].

There was a significant association between stress level and presence of TMD. This finding has mirrored that of multiple other studies [45–48].

Our study has shown that the higher number of hours spent on the phone per day, was associated with higher depression, anxiety and stress scores and higher TMD severity score. In fact, studies have shown that cell phone use is one of the variables associated with the general health of medical students, affecting it negatively [49–51]. Moreover, ergonomic factors related to workplace environment are risk factors for developing neck pain [52]. Effective ergonomic interventions can reduce neck-shoulder pain [53] and predefined activities can help limiting the overuse of cellphones, thus improving sleep quality [51] and potentially reducing anxiety-related behavior and bruxism.

### Results explanation

The probable association between stress, anxiety and temporomandibular joint disorder is that psychological factors are able to produce oral parafunctional habits [45], and that they are associated with a lower pressure pain threshold, affecting masticatory muscle tenderness [54]. The Lebanese population, specifically within the 30–40 years old age group can be considered a war generation having witnessed 15 years of civil war and unrest and thus maybe more prone to anxiety, stress, depression or even mental disorders [55]. Some of these disorders remains underdiagnosed as a social stigma exists towards mental disorders [56], and those seeking treatment for it [57].

### Practical implications and directions for future research

Earlier screening of TMD and thus early intervention with cognitive behavioral therapy leads to better results [58]. Physicians should counsel and educate patients on good oral habits, lifestyle modifications (less hours on a screen, phone …) and if needed refer to dentists as well as screen and treat underlining associated anxiety and depression. TMD may need the collaboration between multiple health specialists, including otolaryngologists, dentists, physiotherapists, and oral and maxillofacial surgeons [59]. High quality studies are needed to better define the use of medications, and better understand the risks and benefits of any drug used [60]. In addition, new diagnostic imaging techniques must be explored to further evaluate the relationship between tinnitus and TMD [61].

### Limitations

An attrition bias is possible because of the refusal rate. A selection bias is also possible since the sample is not representative of the whole population. Information bias might also be present since some questions might be over- or underestimated by the participants. In addition, to better evaluate the association between stress, anxiety and temporomandibular joint disorder, the latter should be classified into its different subtypes [9]. Temporomandibular Disorders (TMDs) is a general term that includes a group of clinical conditions affecting the temporomandibular joint, the masticatory musculature and associated head and neck musculoskeletal structures. Masticatory Myofascial Pain refers to the myogenic component of the disease which is considered to be the most prevalent. The present study did not segregate between the two major subgroups of TMD: a myogenic facial pain (MFP) group and a TMJ internal derangement (TMJID) group. The primary differentiation between the 2 subgroups is based on meniscus displacement present with TMJID patients and this was not identified in our study which relied primary on anamnesis rather than on physical examination. While most of the patients sampled in our study likely belong to the MFP group, a TMJID component cannot be ruled out based on history alone. It is in the light of this distinction that our results must be interpreted. Previous authors [24] have shown that anxiety scores in TMD with muscle disorder and TMD with joint disorder were the same, whereas the prevalence of depression was higher in TMD with muscle disorder than TMD with joint disorder. This resonates with our study results showing a high prevalence of depression likely indicating a comorbidity of the MFP subtype of TMD represented in our population. Another limitation resides in the fact that the scales (except PHQ-9, GAD-7 and BDS-22) have not been validated in Lebanon. Last and not least, since our study is a cross-sectional one, the association and correlation between the variables does not always mean causation.

## Conclusion

This study has shown that TMD remains largely underdiagnosed in the Lebanese population and that it correlates well with anxiety, stress, depression and bad oral and lifestyle habits. We need to raise awareness among the population and health professional alike to better screen for and treat TMD.

## Data Availability

All data generated or analyzed during this study are not publicly available to maintain the privacy of the individuals’ identities. The dataset supporting the conclusions is available upon request to the corresponding author.

## Abbreviations

TMD: Temporomandibular joint disorder
TMJ: Temporomandibular joint
WHO: World Health Organization
GAD: Generalized anxiety disorder
BDS22: Beirut Distress Scale
SPSS: Statistical Package for Social Science
